# Therapeutic Implications of Biomolecular Corona on Lipid Nanoparticle‐Based Gene Delivery Systems

**DOI:** 10.1002/smsc.202500206

**Published:** 2025-07-09

**Authors:** Antonietta Greco, Claudia Corbo

**Affiliations:** ^1^ Department of Medicine and Surgery BioNanoMedicine Center University of Milano‐Bicocca Via R. Follereau 3 20854 Vedano al Lambro MB Italy; ^2^ IRCCS Istituto Ortopedico Galeazzi Via Cristina Belgioioso 173 20157 Milano Italy

**Keywords:** biodistribution, biomolecular corona, biomolecular corona engineering, lipid nanoparticles, nucleic acid delivery

## Abstract

Lipid nanoparticles (LNPs) are widely used in drug delivery due to their low toxicity, excellent biocompatibility, and ability to facilitate endosomal escape. A critical factor influencing the in vivo behavior of LNPs is the formation of a biomolecular corona (BC) on their surface. This layer of biomolecules affects key biological processes such as targeting, absorption, distribution, metabolism, and clearance. Gaining a deeper understanding of the BC formation mechanisms is essential for predicting and optimizing the therapeutic efficacy of LNPs. In this perspective, we present recent advances in the characterization, isolation, and functional implications of the BC. We explore how BC formation affects the stability, biodistribution, and targeting capacity of LNPs, and discuss how harnessing this phenomenon could offer a powerful strategy to improve the precision and effectiveness of targeted drug delivery.

## Graphical abstract

This image illustrates the role of the biomolecular corona in lipid nanoparticles (LNPs), highlighting its impact on the stability, immune response, biodistribution, and targeted delivery. Formed after injection, the corona influences how LNPs circulate, interact with the cells, and modulate the immune response. Understanding and manipulating the corona is crucial for advancing nanoparticle‐based drug delivery systems.

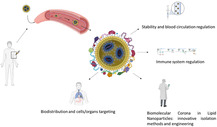

## 1 Introduction

Lipid nanoparticles (LNPs) have emerged as a successful delivery platform for nucleic acid payloads such as siRNA, mRNA, and DNA.^[^ ^1^, ^2^, ^3^ ^]^ The primary challenge in administering naked nucleic acids in the human body is their degradation by nucleases, which reduces their half‐life and activates the immune system.^[^ ^4^, ^5^ ^]^ Encapsulating genetic material into LNPs offers a straightforward solution to overcome these hurdles and deliver the nucleic acids to target tissues.^[^ ^1^ ^]^

Given the clear therapeutic potential of LNPs, numerous recent studies have focused on developing clinically viable lipid‐based formulations by fine‐tuning their physicochemical properties in an attempt to maximize the encapsulation efficiency of genetic materials, enhance particle stability, improve biodistribution and targeting capabilities, and optimize the scalability and reproducibility of the manufacturing process.^[^ ^3^ ^]^

These efforts led to the clinical approval in 2018 of the first LNP‐based siRNA therapeutic, Patisiran (Onpattro), for the treatment of hereditary transthyretin‐mediated amyloidosis. In 2020, due to the urgent need for vaccinations to combat the COVID‐19 pandemic, LNPs enabled the production of the first two SARS‐CoV‐2 mRNA‐based vaccines: Ozinameran (Pfizer‐BioNTech) and Elasomeran (Spikevax or COVID‐19 vaccine Moderna).^[^ ^3^, ^6^, ^7^, ^8^ ^]^ The LNP delivery system technology was essential for the success of these vaccines.^[^ ^9^ ^]^ A key component of this successful technology is the use of ionizable lipids, molecules that contain an amino group with a dissociation constant (pKa) typically ranging between 6 and 7. This pKa range induces LNPs to remain neutral at physiological pH, while becoming positively charged in acidic conditions. Therefore, by using a low pH during LNP formulation, the positively charged amino groups of ionizable lipids readily interact with the negatively charged phosphate groups of nucleic acid cargo. This electrostatic interaction facilitates the formation of an electron‐dense core composed of tightly packed ionizable lipids and nucleic acids.^[^ ^6^ ^]^

Moreover, this pH‐sensitive nature of ionizable lipids is crucial for triggering endosomal escape, a key step in ensuring the efficient intracellular delivery of nucleic acids. Indeed, when injected at physiological pH, LNPs remain neutral, minimizing toxicity and enabling prolonged circulation in the bloodstream. Upon cellular uptake, LNPs are trafficked into endosomes (acidic intracellular compartments) where the drop in pH reionizes the lipid components, rendering them positively charged. These positively charged lipids interact with the negatively charged endosomal membranes, disrupting their integrity and promoting the release of nucleic acids into the cytoplasm, where they can exert their intended biological functions, such as initiating protein translation, as in the case of mRNA vaccines.^[^ ^6^, ^10^ ^]^

In summary, after vaccine injection, innate immune cells internalize LNPs at the administration site. The mRNA is released into the cytosol via pH‐triggered endosomal escape, where it is translated into antigens and presented to CD4 + T helper cells. This stimulates the release of cytokines and chemokines, driving an antigen‐specific immune response.^[^ ^11^ ^]^ The COVID‐19 pandemic paved the way for the widespread use of LNPs worldwide. Currently, LNPs are being extensively investigated to develop innovative vaccines and new therapeutic formulations with a plethora of applications. Indeed, a search in the Scopus database (January 2025) using the term ‘lipid nanoparticles’ revealed a total of 1694 publications in the period from 2015 to 2019, and a total of 4300 in the years 2020–2024, with a notable increase in publications, exceeding at least 100 annually, and reaching 1400 manuscripts in 2024 alone.

Despite the encouraging results obtained with LNPs, no single system is perfect: the main downsides of LNPs include the difficult prediction of their biological fate and pharmacokinetics,^[^ ^8^ ^]^ their low cell and organ targetability, and the discrepancy between in vitro and in vivo results.^[^ ^3^, ^12^ ^]^ These issues arise because, depending on the administration route, LNPs interact with hundreds of biomolecules present in biological fluids (chiefly proteins and lipids, but also nucleic acids and electrolytes), resulting in a coating known as biomolecular corona (BC). The BC exhibits dynamic characteristics: the molecules tightly bound to the nanoparticles form the ‘hard corona’, while the biomolecules on the outer part constitute the ‘soft corona’. The soft corona is loosely associated with both the hard corona and other biomolecules, and it is constantly exchanged with the molecules faced during the nanoparticles’ journey in the biological environment.^[^ ^13^, ^14^ ^]^ Different methods have been developed and optimized to characterize the hard corona,^[^ ^15^, ^16^ ^]^ while only recently, techniques such as field flow fraction^[^ ^17^ ^]^ or photoaffinity‐based chemo–proteomics,^[^ ^18^ ^]^ have been proposed to isolate and study the soft corona.^[^ ^19^ ^]^

The BC is influenced by the physicochemical characteristics of LNPs such as lipid composition, size, and charge. Typically, LNPs consist of four components: 1) ionizable cationic lipids, which enable the encapsulation of nucleic acids through electrostatic interactions at low pH and facilitate endosomal escape under acidic pH conditions, 2) PEGylated lipids, which help to reduce the immune system's recognition of the formulation; 3) Helper lipids, which enhance the LNP's stability and transfection activity; and 4) cholesterol, which affects the LNP's rigidity, cellular uptake, and payload release.^[^ ^6^ ^]^

The combination of these elements have been investigated by many research groups to improve LNP delivery performances and modulate the composition of the BC.^[^ ^1^, ^7^, ^20^, ^21^, ^22^ ^]^ Additionally, the type, origin, concentration, pH, and temperature of biological fluids strongly influence BC formation.^[^ ^3^, ^23^ ^]^ The composition of the BC may vary between individuals depending on the physiological and pathological conditions, as well as demographic factors such as age, sex, and ethnicity, giving rise to the emerging concept of personalized BC.^[^ ^14^, ^19^ ^]^

In this regard, a recent study demonstrated that the BC composition and LNP transfection efficacy differed in plasma from lean and obese mice models. The results showed a correlation between high‐density lipoprotein (HDL) in the BC and LNP efficacy.^[^ ^7^ ^]^

A notable example is provided by Ju et al. who demonstrated that PEG‐coated mesoporous silica nanoparticles (≈100 nm) exhibit significant donor‐dependent variability in the composition of their BC. This variability led to differential interactions with immune cells, highlighting the influence of individual physiological factors on BC formation.^[^ ^24^ ^]^

These findings further underscore the importance of considering patient‐specific physiology, and consequently, personalized BC, when designing drug delivery systems. Recently, Wu and colleagues studied the BC formed on polystyrene nanoparticles in both healthy mice and diseased models, including diabetes and colitis. Their findings revealed that the BC derived from diseased mice was enriched with proteins associated with enhanced intestinal absorption, in contrast to the BC from healthy mice. This shift was favorable for the treatment of diabetes but inadequate for colitis treatment.^[^ ^25^ ^]^ Yu and colleagues demonstrated that the BC formed on PEG‐transferrin‐modified nanoparticles using plasma from patients with nonsmall cell lung cancer significantly reduced the cellular uptake of the nanoparticles, a phenomenon not observed in plasma from healthy individuals.^[^ ^26^ ^]^

These results, among others, underscore the importance of evaluating how pathological conditions can alter the composition of the BC, potentially impacting the therapeutic efficacy and suitability of nanoparticle‐based drug delivery systems.

The BC may well be considered a surface modification of the LNPs because it can affect their physicochemical characteristics and stability, half‐life, targetability, biodistribution, and immune system recognition. Therefore, the modulation of BC represents a real opportunity to improve LNP's efficacy and regulate their biodistribution.^[^ ^27^ ^]^ This has been clearly demonstrated with Onpattro which, when administered intravenously, interacts with the serum protein apolipoprotein E capable of guiding LNPs toward low‐density lipoprotein receptors expressed on hepatocytes’ surface, thereby achieving liver selectivity.^[^ ^1^, ^3^, ^28^ ^]^

**Figure 1:**
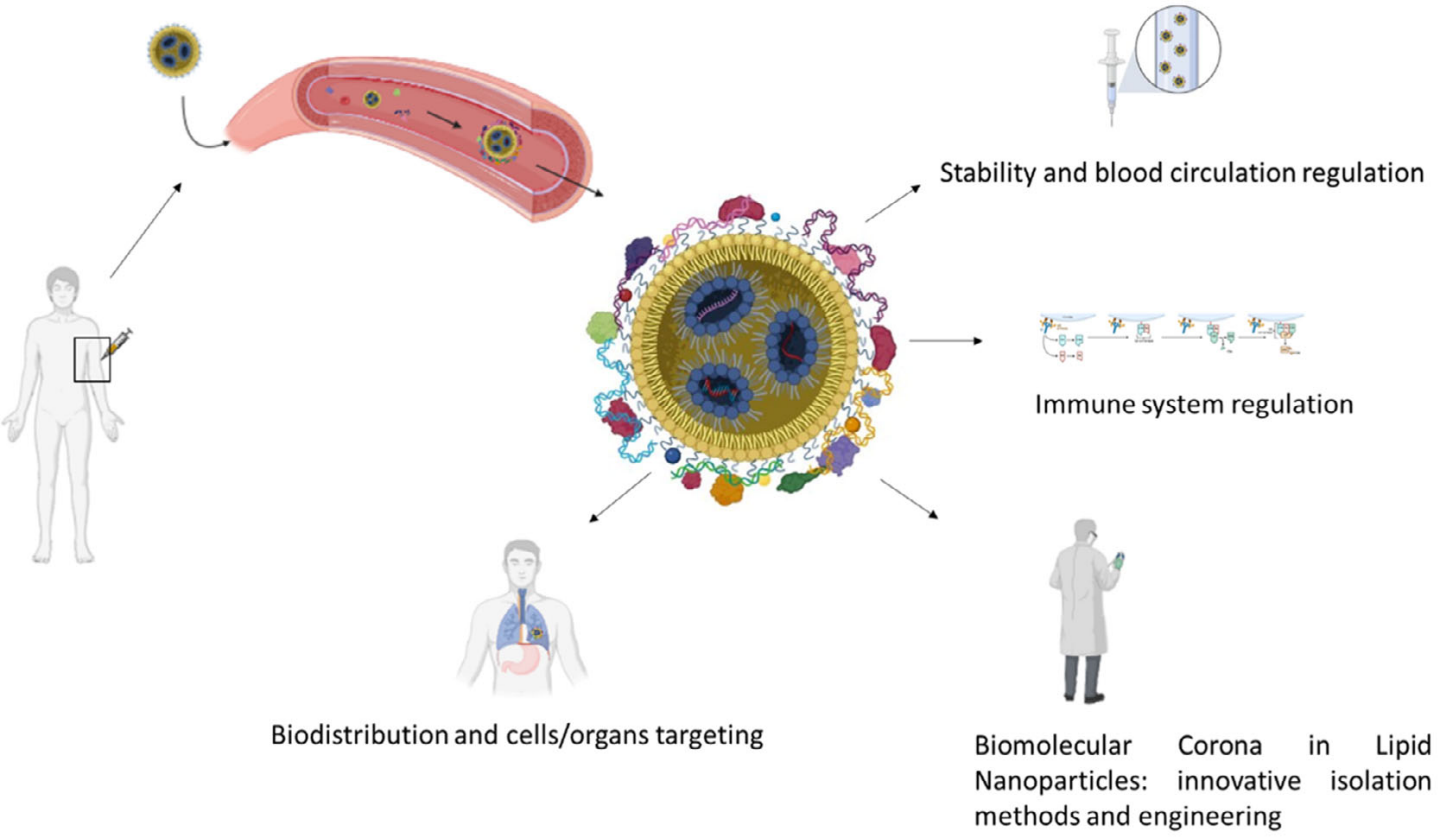
Summary of the themes discussed in this manuscript (Created in https://BioRender.com).

Given the growing development and application of LNPs for nucleic acid delivery, and the key role of BC, this perspective examines the impact of BC on LNPs, pointing out possible strategies to exploit BC for improving the therapeutic performance of LNPs (**Figure** **1**).

Regarding the administration route, LNPs were originally designed and optimized for systemic administration to enable efficient delivery of nucleic acids. In contrast, exploration of alternative administration routes, such as oral, intranasal, or pulmonary delivery, has been relatively limited.^[^ ^29^ ^]^ Among these, oral delivery remains especially challenging due to the presence of several physiological barriers in the gastrointestinal tract, including acidic pH, enzymatic degradation, and the mucus layer. Indeed, although some studies have reported varying degrees of success with orally administered LNPs,^[^ ^29^ ^]^ comprehensive data on their interactions with mucins, the key structural components of mucus, are still lacking.^[^ ^29^, ^30^ ^]^ Moreover, the understanding of which properties of nanoparticles favor mucosal diffusion and systemic uptake, creating an effective oral drug delivery system, is a subject of intensive research. Despite many investigations and recent elucidation on the in vitro/in vivo discrepancies in the nanoparticles’ efficacy due to the BC formation, there is still no clear scientific consensus on the optimal design for effective oral delivery systems.^[^ ^30^ ^]^

Given these challenges, this perspective primarily focuses on the LNPs developed for systemic nucleic acid delivery, specifically referring to its administration via the bloodstream (i.e., parenteral injection), where design parameters, biological interactions, and therapeutic outcomes are more comprehensively characterized.

## 2 BC in LNPs: Innovative Isolation Methods

The BC plays an essential role in the LNPs’ identity and activity. The traditional approach for extracting the BC of LNPs involves incubating the nanoparticles with biological fluids, followed by isolating, separating, and analyzing the BC. In more detail, the LNPs’ incubation conditions in the biological milieu should be selected according to the studied disease, as well as the administration route and modality. After BC formation, it is essential to study how the cargo is released and how LNPs’ stability, targeting, and cellular uptake are influenced.^[^ ^1^, ^3^, ^14^, ^31^, ^32^ ^]^

**Figure 2:**
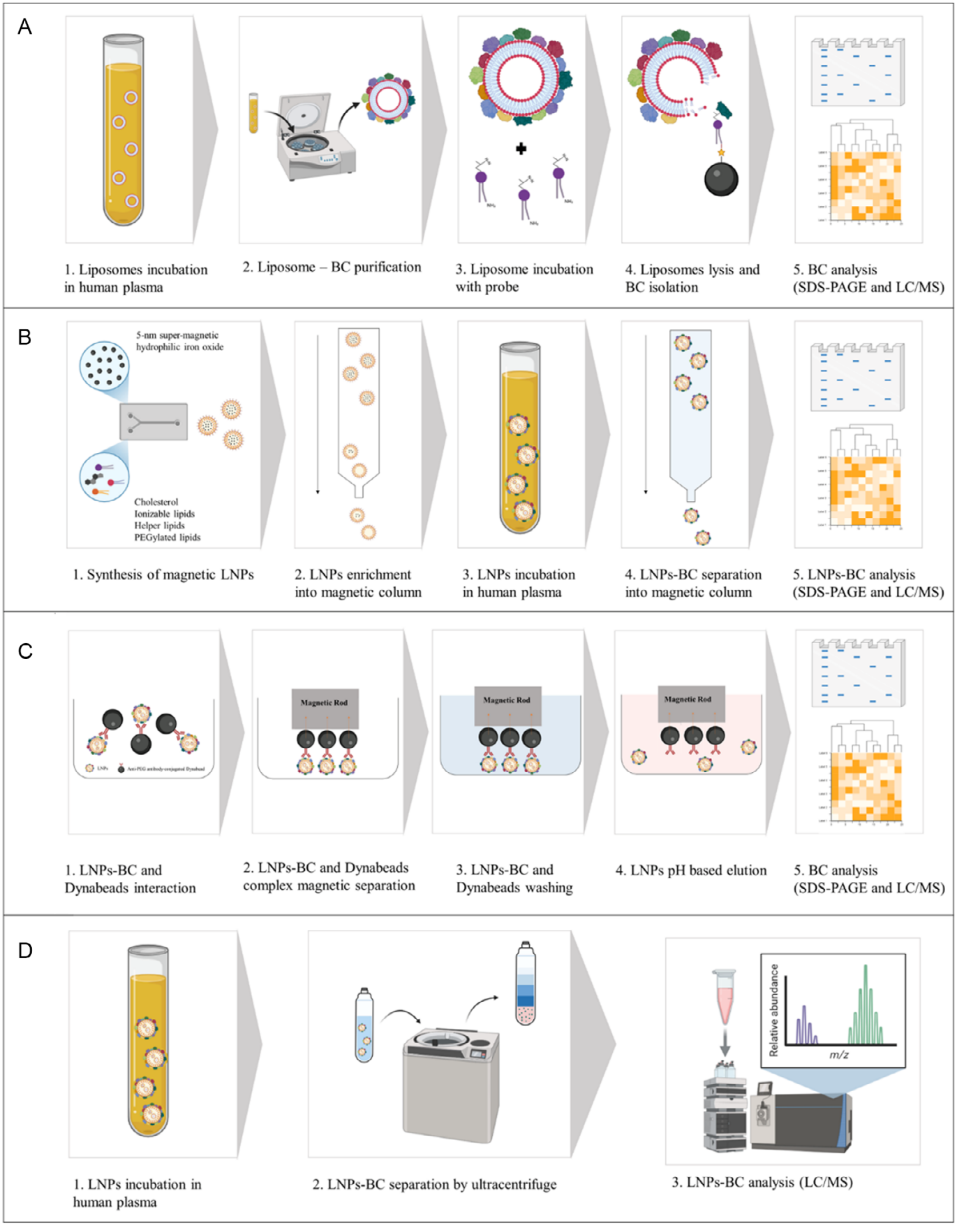
Illustration of the innovative methods for BC isolation from the LNPs. A) BC identification using photoaffinity‐based chemoproteomics: Liposomes were first exposed to plasma samples and then purified by centrifugation; the photoaffinity lipid probes (structurally similar to phospholipids), when incubated with LNPs, interacted with the BC; the photoprobe enabled the monitoring and study of the dynamic interaction between the nanoparticles and proteins; proteins were analyzed by gel electrophoresis and LC–MS.^[^ ^18^ ^]^ B) Synthesis of magnetic LNPs: loading of 5 nm supermagnetic hydrophilic iron oxide nanoparticles; magnetic‐LNPs were purified through a magnetic column placed into a magnetic separator; iron‐enriched magnetic LNPs were incubated in human serum for 1 h at 37 °C to form the BC and then purified using a magnetic column; the recovered LNPs–BC were analyzed by SDS–PAGE and LC–MS.^[^ ^1^ ^]^ C) Anti‐PEG antibody‐conjugated magnetic beads (Dynabeads) captured LNPs–BC in a 96 multiwall; LNPs–BC and Dynabeads complexes were magnetically separated and, to remove nonspecifically bound biomolecules, a series of washing steps were performed. The LNPs–BC were eluted by alternating the pH and BCs were analyzed by LC–MS.^[^ ^7^ ^]^ D) LNPs were incubated in plasma samples to form the BC. For LNPs–BC complex isolation, a continuous density gradient ultracentrifugation was applied; proteins were identified by LC–MS.^[^ ^35^ ^]^ The illustration was generated with BioRender (https://BioRender.com).

The separation of the intact BC from the unbound biomolecules is not trivial. The conventional BC isolation methods include size‐exclusion chromatography or ultracentrifugation; however, these techniques do not guarantee effective separation of the LNPs from the numerous lipoproteins and extracellular vesicles naturally present in the biological fluids because these entities are extremely similar in terms of physicochemical characteristics.^[^ ^1^, ^3^, ^7^, ^33^ ^]^ In addition, LNPs are soft nanomaterials. Therefore, traditional approaches could result in artefacts derived from the LNPs’ aggregation with biomolecules, thereby complicating the differentiation between unbound biomolecules and those adsorbed on the LNPs’ surface. The lack of effective high‐throughput BC isolation procedures for soft materials has driven the development of alternative techniques. One approach consists in the use of a photoaffinity lipid probe able to capture the hard BC of a liposome, allowing the removal of the unbound BC proteins and offering the possibility to follow and study the dynamic interaction between the nanoparticles and proteins by exploiting the spatiotemporal resolution of the activated photoprobe (**Figure** **2**A).^[^ ^18^ ^]^ Recently, Francia et al. proposed a magnetic separation method that allows a more accurate characterization of the LNPs’ BC compared to traditional methods. Briefly, they created magnetic LNPs by loading 5 nm superparamagnetic hydrophilic iron oxide nanoparticles within the LNPs. These magnetic LNPs could then be isolated from the unbound proteins in the serum, using a magnetic separation procedure that preserves the integrity of LNP–BC complexes. This straightforward and effective approach permitted identification of a unique BC, specifically enriched in certain proteins, not well captured by other techniques. Indeed, among the formulated lipids, the ionizable ones critical for the nucleic acid delivery, greatly influence the level of BC adsorption (Figure 2B).^[^ ^1^ ^]^ Although this method offers advantages for BC investigation, it is important to consider that including magnetic nanoparticles within LNPs could alter their physicochemical properties. As will be discussed in Section 3.4, the nanoparticle properties such as hydrophobicity play a significant role in determining the pattern of the adsorbed molecules, thereby influencing the overall composition of the BC.^[^ ^34^ ^]^ These alterations could impact LNP stability, biodistribution, and cellular uptake. Therefore, while magnetic separation offers a valuable method for BC analysis, potential modifications introduced by the magnetic nanoparticles must be carefully evaluated to ensure accurate interpretations of results.

Another interesting automated high‐throughput corona isolation method was proposed by Liu et al. They first conjugated Dynabeads with antibodies capable of interacting with free biomolecules; both Dynabeads and LNPs were incubated using an antibody: mRNA (wt:wt) ratio of 1. Afterward, Dynabeads were extracted using magnetic rods, washed, and separated from LNPs as particles with an intact BC through basic pH elution (Figure 2C). This study allowed to explore the key role of HDL on the LNPs efficacy. Indeed, authors found that the most effective LNP–BC complexes were enriched with HDL and compared to the commonly used BC marker apolipoprotein E. The HDL content of the BC was a better predictor of in vivo activity. The method validates HDL as a source of apolipoprotein E to improve the LNPs’ therapeutic efficacy by controlling BC composition.^[^ ^7^ ^]^ Another innovative method has recently been proposed by Voke and colleagues, who developed a quantitative, label‐free mass spectrometry‐based proteomics approach to characterize the LNPs’ BC. Specifically, for BC–LNP complex isolations, they applied continuous density gradient ultracentrifugation, while for protein identification (normalized to protein composition in the biofluid alone) was performed using mass spectrometry analysis (Figure 2D). This approach helps avoid artefacts introduced by the presence of endogenous nanoparticles in human biofluids, (e.g., extracellular vesicles) and allows the quantification of proteins consistently enriched in the LNP BC, including alpha‐2 macroglobulin, C‐reactive protein, and vitronectin. The authors also explored the impact of the BC on cellular uptake of LNPs and on mRNA expression using HepG2 human liver cells, revealing that increased LNP uptake was not directly correlated with a higher mRNA expression, possibly due to BC‐induced lysosomal trafficking of LNPs. These results further support the impact of the BC on LNP fate and highlight the need to consider the BC in the design of LNP‐based therapeutics.^[^ ^35^ ^]^ A schematic representation of the methods described above is illustrated in Figure 2.

Once isolated, the BC composition is usually analyzed by liquid chromatography–mass spectrometry (LC–MS), which has become the gold standard technique to quantify and identify the individual biomolecules forming the corona.^[^ ^3^, ^36^ ^]^ The effect of BC on the nanoparticles is assessed using techniques such as dynamic light scattering, transmission electron microscopy, and nanoparticle tracking analysis. A combination of these methods provides comprehensive information about the LNPs’ BC.^[^ ^3^ ^]^

To date, proteins are the most extensively characterized biomolecules forming the BC on nanoparticles.^[^ ^3^ ^]^ Accordingly, **Table** **1** lists key proteins commonly identified in the BC of LNPs, along with their primary biological functions and potential impact on LNP's behavior and performance.

**Table 1:** Commonly adsorbed proteins identified in the LNPs’ BC and their implications on biological function.

Protein	Biological Function	Impact on LNPs	Reference
α‐2‐macroglobulin	Humoral defense by intercepting and binding exogenous peptides and particles	Immune activation and mononuclear phagocyte system clearance	[3, 7, 35, 80, 81]
Apolipoprotein E	Enhance the interactions with low‐density lipoprotein receptors for lipid transport and metabolism	Immune evasion, liver sequestration, and blood–brain barrier penetration	[3, 7, 80, 81, 82]
C‐reactive protein	Complement pathway activation	Immune activation and mononuclear phagocyte system clearance	[35, 80, 83]
Immunoglobulin	Humoral immunity by eliminating pathogens	Immune activation and mononuclear phagocyte system clearance	[3, 7, 80, 81]
Serum albumin	Most abundant protein of plasma essential for homeostasis	Immune evasion and vasculature targeting	[3, 7, 80, 81, 84]
Vitronectin	Cell adhesion and spreading factor	Immune activation and mononuclear phagocyte system clearance	[7, 35, 80, 81]
Clusterin	Enhance the lipid transport, cell adhesion, and immune system regulation	Reduced cellular uptake in human macrophage‐like cells	[3, 7, 49, 80, 85]
Fibrinogen‐like 2	Blood clotting	Immune activation, mononuclear phagocyte system clearance, and induction of coagulation	[7, 81, 86]
Serotransferrin	Iron transport throughout the body	Increased blood circulation time	[3, 7, 87, 88]
Complement C1, C3	Innate immune surveillance	Immune activation and mononuclear phagocyte system clearance	[7, 81, 89]

## 3 How BC Affects LNPs Delivery

**Figure 3:**
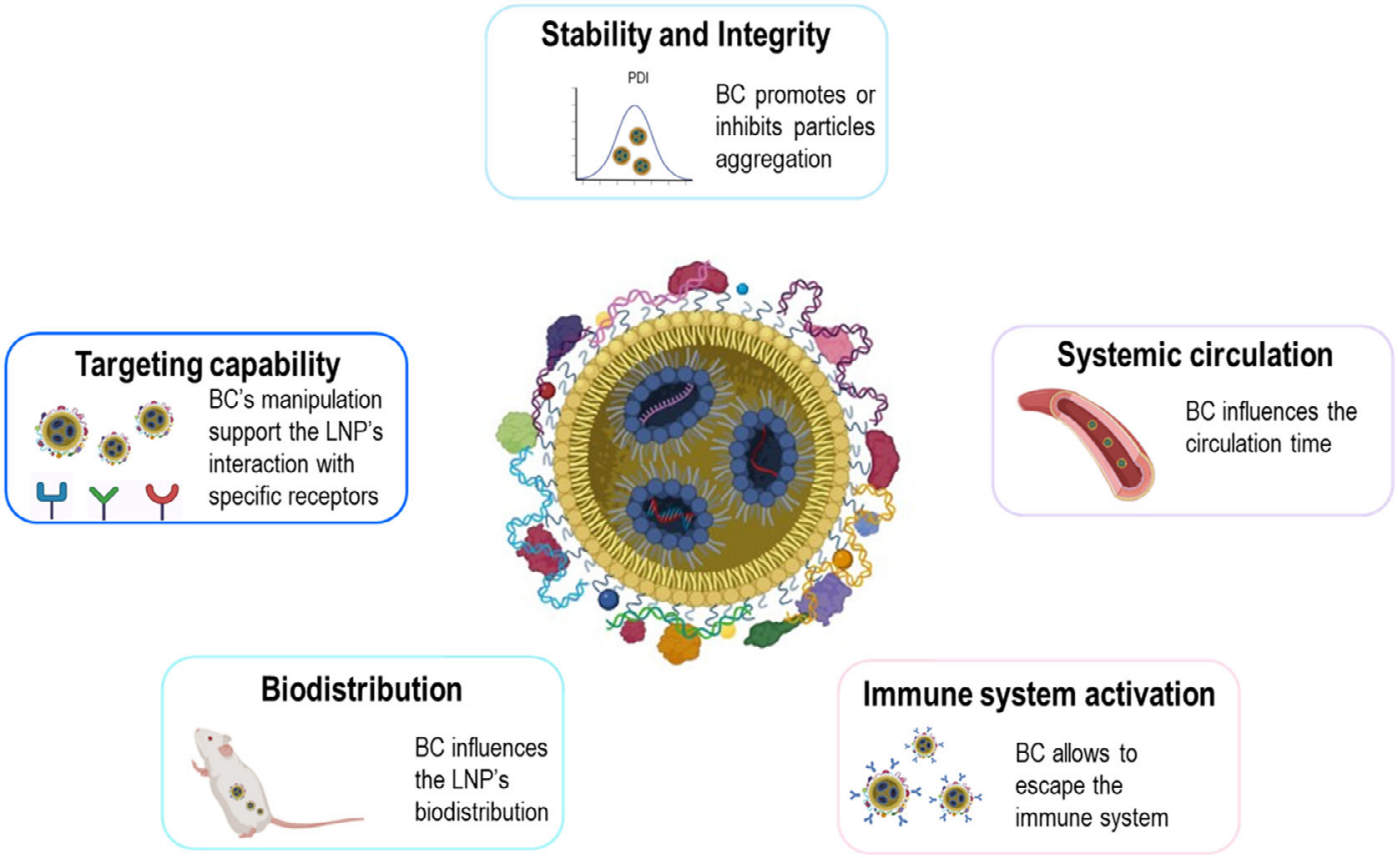
Graphical representation of BC's impact on LNP's stability, integrity, systemic circulation, immune system activation, biodistribution, and targeting capability (Created in https://BioRender.com).

LNP efficacy hinges on the meticulous regulation of physicochemical characteristics such as size, charge, rigidity, and morphology. However, these properties change when LNPs are exposed to biological fluids. Therefore, understanding the impact of the BC on LNP's stability, integrity, systemic circulation, immune system activation, biodistribution, and targeting capability is crucial (**Figure** **3**).^[^ ^3^ ^]^

### 3.1 LNPs Stability and Integrity

Ensuring the stability and integrity of LNPs is essential for maintaining their functional properties. Recent studies have shown that the BC can either promote or inhibit particle aggregation by modifying surface characteristics.^[^ ^37^ ^]^ This can affect the LNP circulation time, biodistribution, targeting, release profile, macrophage recognition, toxicity, and uptake kinetics.^[^ ^3^ ^]^ For instance, electrostatic interactions between the cationic nanoparticles and anionic proteins in the bloodstream can trigger particle agglomeration, leading to reduced circulation times, decreased accumulation in target tissues, diminished cellular uptake, and impaired endosomal escape.^[^ ^38^, ^39^ ^]^ The BC can also impact the integrity of nanoparticles, potentially inducing the leakage of fluorophores and/or radiotracers.^[^ ^3^ ^]^ This instability in biological fluids may help explain the failure of certain LNP formulations to deliver genetic material effectively in vivo.^[^ ^3^, ^40^ ^]^

### 3.2 LNP's Biodistribution and Immune System Recognition

The biodistribution, immune system recognition, and blood clearance capability strongly influence the LNP's fate within the organism. These pharmacokinetic parameters are tightly connected with the physicochemical properties of LNPs, which can be modified upon biomolecule adsorption on their surfaces.^[^ ^41^ ^]^ Indeed, the BC formation can trigger coagulation cascades and promote the binding of opsonins, such as immunoglobulins, complement factors, and unfolded fibrinogen, leading to the recognition and rapid clearance by the mononuclear phagocyte system.^[^ ^42^, ^43^ ^]^ This immune response is known to hinder the efficient nanoparticle delivery to the target organs and reduce its therapeutic efficacy.^[^ ^3^, ^44^, ^45^ ^]^

To address this issue and try to minimize the particle clearance from the bloodstream, it is possible to modify the LNP's lipid composition.^[^ ^3^ ^]^ Another strategy to escape the immune system recognition is by altering the particle surface, either during or after the synthesis, with hydrophilic polymers that have a neutral charge at physiological pH. The most frequently used polymer is polyethylene glycol (PEG), which reduces macrophage uptake and extends nanoparticle's blood circulation time.^[^ ^46^, ^47^ ^]^ Indeed, it is known that proteins present in the corona of PEGylated nanomedicines (e.g., apolipoprotein J) are associated with reduced opsonization and prolonged systemic circulation time.^[^ ^48^, ^49^, ^50^ ^]^ While PEGylation can confer longer circulation time, it can simultaneously reduce cellular uptake and hinder endosomal escape.^[^ ^51^ ^]^ Moreover, the widespread use of PEGylation has led to a poorly characterized anti‐PEG immune response and highly variable outcomes in clinical trials.

As a result, this approach is gradually being replaced by novel synthetic peptides, originally developed to selectively modify proteins, which are now being applied more broadly in therapeutic strategies for both structural enhancement and microenvironmental modulation.^[^ ^4^ ^]^ For example, poly(N‐vinylpyrrolidone) is a hydrophilic polymer that, when conjugated to nanoparticles, can enhance their biocompatibility without adversely affecting cell viability. Interestingly, poly(2‐oxazolines) are highly biocompatible polymers capable of prolonging the blood circulation time of drug delivery systems while evading immune detection. This is because poly(2‐oxazolines) are less immunogenic than PEG and minimize the interaction with proteins typically recognized from the immune system (e.g., components of the complement system, immunoglobulins, and fibrinogen). Hydrophilic polycarbonates are biocompatible and biodegradable synthetic amphiphilic polymers. Due to their features, they can be easily included within self‐assembly nanostructure and potentially control their release profile. Moreover, the presence of functional groups on the hydrophilic polycarbonates facilitates the superficial modifications of drug delivery systems.^[^ ^4^, ^52^, ^53^ ^]^

### 3.3 LNP's Delivery and Targeting

The targetability of LNPs is essential to ensure an efficient therapy. Usually, the targeting is achieved by the physical or chemical LNP surface conjugation of certain ligands able to recognize specific receptors. For example, Breda and colleagues conjugated a fraction of the anti‐CD117 antibody on the LNP surface for the delivery of mRNA. Results showed that LNPs were able to efficiently target hematopoietic stem cells and induce durable genome editing, bringing a near‐complete correction of the hematopoietic sickle cells.^[^ ^54^ ^]^ Nevertheless, it is known that BC could mask the targeting ligands on the LNP surface, limiting the interaction with the corresponding receptors. On the other hand, several examples have reported that BC can be leveraged to enhance interactions with specific cell receptors and modulate the LNP's cellular uptake and the tissue/organ accumulation.^[^ ^8^, ^28^ ^]^ To understand how the BC affects the LNP's fate, it is crucial to identify the proteins responsible for the cell‐LNP interaction and define their orientation and conformation.^[^ ^1^, ^3^ ^]^ For this purpose, immunolabeled gold or quantum dots have been employed to investigate these aspects through electron microscopy and flow cytometry, respectively.^[^ ^19^, ^55^, ^56^ ^]^

Walkey et al. conducted an interesting study in which they analyzed the BC of a library of 105 gold nanoparticles obtained after their incubation in whole blood. The authors discovered that adsorbed biomolecules behave as a “fingerprint,” which allowed to accurately predict the nanoparticles–cells interactions.^[^ ^57^ ^]^ A similar approach was applied to a library of 16 LNPs to investigate the relationships between physicochemical properties, BC fingerprints, and cellular uptake. The study revealed that only a small subset of proteins within the BC significantly enhanced the interaction of LNPs with HeLa cells.^[^ ^58^ ^]^ The modulation of the lipid composition of LNPs is another strategy applied to control targeting. In this regard, Chen et al. reported, for the first time, how to adjust the systemic tropism for CRISPR‐Cas gene editing known as selective organ targeting (SORT). Briefly, the researchers found that progressively increasing the amount of a cationic lipid, referred to as the SORT lipid, led to a shift in LNP targeting from the liver to the spleen, and eventually to the lungs. This organ‐specific distribution was linked to the enrichment of distinct components within the BC.^[^ ^20^ ^]^

Similarly, Dahlman and colleagues administered 30 different LNP formulations in mice and correlated their chemical properties with tissue‐specific delivery profiles, further highlighting the interplay between nanoparticle composition, BC formation, and biodistribution.^[^ ^59^ ^]^ In another study, Sago et al. evaluated how 250+ LNPs with different chemical characteristics could deliver functional mRNA to multiple cell types in vivo.^[^ ^60^ ^]^ Recently, Dilliard and colleagues demonstrated how to formulate lung SORT LNPs by using a panel of quaternary ammonium lipids capable of varying the BC composition. They found that the major proteins into BC were associated with the delivery to lungs, concluding that LNP distribution is affected by a pool of proteins rather than a single molecule.^[^ ^61^, ^62^ ^]^ This finding was further corroborated by Qiu et al. who demonstrated that LNP lung targeting arises from the synergistic interaction of various proteins within the BC.^[^ ^21^ ^]^

In the context of LNP‐based vaccines, BC could contribute to specific interactions with immune cells and modulate the immune response. The general idea is to exploit the immunomodulatory role of proteins within the BC to enhance the interaction between LNPs and immune cells.

One approach involves formulating nanoparticles that trigger fibrinogen unfolding, exposing the C‐terminus of the gamma chain, which enhances LNP's interaction with Mac‐1 receptor. This interaction activates the NF–kB pathway, leading to the release of inflammatory cytokines and promoting the vaccine's immune response.^[^ ^8^, ^63^ ^]^ Building on this concept, Amici and colleagues recently proposed integrating the BC into LNPs as a strategy for developing next‐generation gene‐based vaccines.^[^ ^22^ ^]^

### 3.4 Engineering the BC

Part of the success of LNPs in clinical translation is linked to the BC formation. Indeed, engineering the BC can enhance the efficacy and safety of the LNPs as therapeutics or vaccines entities.^[^ ^8^, ^19^ ^]^

The main strategies of BC engineering include the regulation of the physicochemical properties of nanoparticles (e.g., size, shape, and surface roughness), the modification of the nanoparticle surface via the addition of selected functional groups and/or biological moieties,^[^ ^50^ ^]^ and the use of a precoating of nanoparticles prior to exposure to biological fluids.^[^ ^23^, ^64^ ^]^

As previously reported, the physiochemical characteristics of nanoparticles are crucial for the BC formation. Specifically, the BC evolves more rapidly on smaller nanoparticles compared to larger ones, which, however, can adsorb a greater quantity of proteins.^[^ ^65^, ^66^ ^]^

Therefore, smaller nanoparticles with hydrophilic and neutral surfaces, such as PEGylated ones, can significantly limit the BC formation.^[^ ^3^, ^23^, ^67^ ^]^ Indeed, the hydrophilic and hydrophobic properties of nanomaterials impact protein adsorption onto the nanoparticles’ surface.^[^ ^34^ ^]^ Hydrophilic surfaces tend to reduce protein binding by enhancing water solubility and preventing nonspecific interactions, whereas hydrophobic surfaces promote protein adsorption due to favorable interactions with the hydrophobic regions of proteins.^[^ ^3^, ^34^, ^68^ ^]^ Moreover, specific pools of proteins are known to preferentially interact with hydrophilic (e.g., haptoglobin) or hydrophobic surfaces (e.g., vitronectin). This property is strategically exploited to modulate the composition of the BC, allowing for the tailored design of nanoparticles with desired biological interactions.^[^ ^34^ ^]^ A valuable strategy for modulating the BC composition is the use of hydrophilic motifs such as PEG,^[^ ^3^, ^69^ ^]^ as described in the Section 3.2. PEG is a hydrophilic, biocompatible polymer^[^ ^52^ ^]^ widely used in various applications due to its ability to confer a ‘stealth effect’ to nanoparticles by preventing opsonization and minimizing protein adsorption on the nanoparticle's surface.^[^ ^3^ ^]^ In LNP formulations, PEG plays a crucial role by enhancing its stability in biological fluids, prolonging circulation half‐life, and improving in vivo distribution.^[^ ^70^ ^]^ Interestingly, the length of PEG chains has a significant impact on the performance of nanoparticles, influencing their biodistribution overall efficacy.^[^ ^3^ ^]^

The shape of nanoparticles also plays a crucial role in BC formation. For example, mesoporous silica nanoparticles with a spherical shape exhibited a thinner, yet complete and uniform corona, whereas rod‐shaped nanoparticles showed a more abundant BC, primarily consisting of a soft corona.^[^ ^71^, ^72^ ^]^

The surface modification of the nanoparticles with selected moieties allows for the natural and tailored adsorption of precise biomolecules that can target LNPs upon administration. For example, the adsorption of retinol on the nanoparticles’ surfaces promotes targeting to hepatic stellate cells in a mouse fibrotic model.^[^ ^73^ ^]^ Similarly, Kim and colleagues demonstrated that trivalent cholesterol enhanced the nanoparticles targeting the myofibroblasts in fibrotic mice.^[^ ^74^ ^]^ An interesting study by Zhang and collaborators showed that nontoxic peptides derived from amyloid beta, when displayed on the nanoparticle surface, selectively bind apolipoproteins and support the overcome of blood–brain barrier, enabling brain delivery.^[^ ^75^ ^]^

The preformation of an artificial BC during the LNP synthesis can contribute to the modulation or limitation of the BC formation when the LNPs are in contact with biological fluids.^[^ ^8^, ^76^ ^]^ Tonigold and colleagues demonstrated that the preformed BC, composed of adsorbed antibodies, efficiently promoted the interaction with the targeted receptor, achieving better outcomes compared to nanoparticles functionalized directly with the same antibody.^[^ ^77^ ^]^ Similarly, nanoparticles precoated with apolipoprotein E were shown to cross the blood–brain barrier^[^ ^78^ ^]^ and contributed to the prolonged systemic circulation time.^[^ ^79^ ^]^

It is important to highlight that an engineered BC can only be utilized if it is robust enough to remain stable once exposed to biological fluids and if it can compete with endogenous ligands for the same receptors. However, as previously noted, the distribution of nanoparticles and their interactions with receptors might result from a combination of corona proteins rather than a single one. Therefore, it is necessary to evaluate the performance of a single‐protein corona in influencing the nanoparticle delivery.^[^ ^19^ ^]^ Further studies would be valuable to clarify whether BC engineering can enhance LNP's targeting and efficacy.

## 4 Conclusion

LNPs have become a milestone technology for gene therapy and vaccines. The BC formation, resulting from a dynamic interaction between LNPs and the biological environment, deeply influences the behavior of nanoparticles in terms of biodistribution, targeting, and stability.^[^ ^3^, ^19^, ^37^ ^]^ Consequently, BC plays and will play a crucial role in the clinical performance of LNPs, influencing biodistribution, immune interactions, and therapeutic efficacy. Addressing these challenges through surface modifications, targeted corona engineering, and personalized nanomedicine strategies will be critical to improve the translational success of LNP‐based therapies. For personalized treatment, future research should focus on corona profiling in patient cohorts to optimize LNP formulations for individual needs. To achieve this, artificial intelligence technologies could be leveraged to better study the BC and unlock the full potential of LNP‐based therapies.

## Conflict of Interest

The authors declare no conflict of interest.
